# Heart failure in autoimmune rheumatic diseases

**DOI:** 10.1007/s00296-026-06101-8

**Published:** 2026-03-26

**Authors:** Yuliya Fedorchenko, Olena Zimba, Mykhailo Fedorchenko, Bekzhan A. Permenov, Burhan Fatih Kocyigit

**Affiliations:** 1 Department of Pathophysiology, Ivano-Frankivsk National Medical University, Ivano- Frankivsk, Ukraine; 2 Department of Rheumatology, Immunology and Internal Medicine, University Hospital in Kraków, Kraków, Poland; 3 National Institute of Geriatrics, Rheumatology and Rehabilitation, Warsaw, Poland; 4 Department of Internal Medicine N2, Danylo Halytsky Lviv National Medical University, Lviv, Ukraine; 5 Department of Therapy, Family and Emergency Medicine, Faculty of Medicine, Ivano- Frankivsk National Medical University, Ivano- Frankivsk, Ukraine; 6 Department of Social Health Insurance and Public Health, South Kazakhstan Medical Academy, Shymkent, Kazakhstan; 7 Department of Cardiac Surgery Anesthesiology and Intensive Care, Heart Center Shymkent, Shymkent, Kazakhstan; 8 Department of Internal Medicine, Khoja Akhmet Yassawi International Kazakh-Turkish University, Turkistan, Kazakhstan; 9 Department of Physical Medicine and Rehabilitation, University of Health Sciences, Adana Health Practice and Research Center, Adana, Türkiye

**Keywords:** Heart failure, Rheumatic diseases, Autoimmune diseases, Rheumatoid arthritis, Systemic lupus erythematosus, Myocardial dysfunction, Inflammation

## Abstract

Heart failure (HF) is an increasingly important cause of morbidity and mortality in patients with autoimmune rheumatic diseases. Despite advances in cardiovascular prevention and treatment, HF incidence continues to rise in this population, driven by chronic systemic inflammation, immune-mediated myocardial injury, microvascular dysfunction, fibrosis, and treatment-related cardiotoxicity. Epidemiological studies consistently demonstrate a markedly increased HF risk across a broad spectrum of rheumatic diseases—including rheumatoid arthritis, systemic lupus erythematosus, systemic sclerosis, idiopathic inflammatory myopathies, ankylosing spondylitis, and primary Sjögren syndrome—often manifesting at younger age and independently of traditional cardiovascular risk factors. Subclinical myocardial involvement is frequent and commonly precedes overt HF, with preserved ejection fraction representing the dominant phenotype, particularly in inflammatory arthritis and systemic sclerosis. Advances in speckle-tracking echocardiography, cardiac magnetic resonance, and circulating biomarkers such as natriuretic peptides and cardiac troponins have enabled earlier detection and refined risk stratification. Although anti-inflammatory therapies, including conventional and biologic disease-modifying antirheumatic drugs, may mitigate HF risk, optimal control of traditional cardiovascular risk factors and cautious use of cardiotoxic agents remain essential.

## Introduction

Heart failure (HF) is a global public health issue as a leading cause of morbidity and mortality across diverse populations [1]. Despite advances in cardiovascular therapies, the incidence of HF continues to rise, particularly among patients with rheumatic diseases [2]. Patients with rheumatic diseases are prone to cardiovascular morbidity and mortality [2]. The risk of HF is doubled in patients with rheumatoid arthritis (RA) [3] and quadrupled in systemic lupus erythematosus (SLE) compared with the general population [4]. A systematic review of 122 studies demonstrated that patients with inflammatory and autoimmune diseases have a significantly higher risk of HF (OR = 1.28; 95% CI 1.05–1.55) [5]. Drug therapies, particularly long-term exposure to hydroxychloroquine, may contribute to cardiotoxicity and excess risk of HF [6].

The pathophysiology of HF in autoimmune rheumatic diseases is multifactorial. Chronic systemic inflammation may trigger endothelial dysfunction and oxidative stress, impairing myocardial perfusion and promoting fibrosis [7]. Cardiotropic autoantibodies may directly target cardiac structures, leading to myocyte damage and autoimmune myocarditis. Disease-specific autoantibodies, unique to each autoimmune rheumatic condition, together with other antibodies directed against myocardial constituents, may induce inflammation and fibrosis, and ultimately lead to the onset of both systolic and diastolic HF [8]. Advanced imaging modalities such as cardiac magnetic resonance (CMR) may aid in detecting early signs of myocardial alterations in patients with rheumatic diseases [9].

Recognizing HF as a major extra-articular manifestation of rheumatic diseases is a clinically important issue [10], requiring a multidisciplinary approach to early detection, prevention, and management by rheumatologists and allied health specialists [11].

This review aims to overview available evidence on mechanisms, early detection, and management of HF in autoimmune rheumatic diseases.

### Search strategy

Following established guidelines for narrative reviews [12], comprehensive literature searches were performed via Medline/PubMed, Scopus, and the Directory of Open Access Journals (DOAJ) up to December 1st, 2025. The following keywords were employed: “heart failure” OR “cardiac dysfunction” AND “rheumatoid arthritis,” “systemic lupus erythematosus,” “systemic sclerosis,” “Sjögren’s syndrome,” “ankylosing spondylitis,” “polymyositis,” “dermatomyositis,” and “rheumatic diseases”. The analysis was restricted to English research reports, reviews, meta-analyses, and case reports addressing HF epidemiology, pathophysiology, diagnostics, and management in autoimmune rheumatic diseases. Conference abstracts, book chapters, and preprints were excluded. To ensure a thorough review, no specific date restrictions were set during the literature search.

### Heart failure in systemic lupus erythematosus

SLE is associated with a markedly elevated cardiovascular risk, including a nearly threefold increase in HF incidence (RR = 2.89; 95% CI 1.63–5.13) [13]. A US-based population study suggested that HF burden is increased across all SLE age groups, with the highest relative risk of HF observed in young males aged 20–24 years (Relative Risk [RR] 65.2, 95% CI 35.3–120.5) [14]. A Danish registry of 3,411 SLE patients demonstrated an elevated 10-year absolute HF risk (3.71% vs. 1.94%) and higher mortality among SLE patients with HF (adjusted Hazard Ratio [HR] = 1.5; 95% CI, 1.08–2.08) [15]. Early-onset HF is frequently associated with myopericarditis and valvular disease, whereas delayed-onset HF is associated with lupus nephritis and arterial hypertension [16]. The Black race independently confers a 1.5-fold increased HF risk [16]. HF in SLE may progress to terminal disease necessitating heart transplantation and post-transplantation immunosuppressive rehabilitation [17].

Specific causes of HF in SLE include antimalarial-induced cardiomyopathy and acute lupus myocarditis [18]. A systematic analysis of 47 cases of antimalarial-induced cardiomyopathy revealed female predominance (89%), a mean age of 56 years, and long-term exposure to antimalarials (13 years) [18]. Congestive HF was reported in 77% of cases, with left ventricular systolic dysfunction in 53%, conduction disturbances necessitating pacemaker implantation in 51%, and mortality in 45% [18]. Dilated cardiomyopathy in SLE is sporadic [19]. An analysis of 10 such cases (mean age 31 years, 9 females) demonstrated that high-dose corticosteroids led to clinical and functional recovery in 9 cases, supporting the role of early steroid therapy as an effective first-line intervention [19].

Available molecular studies provide insight into SLE-related HF. In fact, gene expression analyses have identified 999 differentially expressed genes enriched in Th17 differentiation pathways, with central hub genes HSP90AB1, UBC, NEDD8, RPLP0, and UBB upregulated in failing hearts and correlating with dilated cardiomyopathy [20].

Comparisons with diabetes mellitus indicate that HF incidence in SLE is comparable to that in diabetes mellitus, with US Medicaid data reporting an HF incidence of 6.9 per 1,000 person-years in SLE versus 6.6 in diabetes mellitus [21].

Cardiovascular complications are a major source of morbidity in SLE. In the KORNET registry of 437 SLE patients, 12 (2.7%) developed cardiac involvement over a median 47.6-month follow-up, with a higher incidence in males, those with anti-Sm and anti-Ro positivity [22]. Male gender, older age, anti-Sm positivity, and baseline corticosteroid use were identified as independent predictors of cardiac involvement [22].

Myocardial involvement in SLE is often subclinical and undetected by conventional echocardiography [23–25]. Two-dimensional (2D) and three-dimensional (3D) speckle-tracking echocardiography (STE) identify early left ventricular (LV) dysfunction in SLE patients with preserved LVEF [23]. Peak strain dispersion (PSD) has emerged as a sensitive marker of early LV dyssynchrony [26]. SLE patients present with PSD and elevated LV mass, mostly in severe disease [27]. A systematic review of 9 observational reports has confirmed these results [28].

In a systematic review of case-control studies including 174,442 SLE patients, the frequency of HF was 2.4% in SLE, representing a 3.4-fold higher risk [29]. Lupus patients demonstrated reduced LVEF [29].

Additional analyses have supported the clinical relevance of strain-based metrics [30]. Among 58 SLE patients without overt cardiovascular disease, PSD was significantly higher and LV global longitudinal strain significantly lower compared with 60 healthy controls (both *p* < 0.05) [30]. In a cross-sectional study of 46 Hispanic SLE patients and matched controls, subclinical LV dysfunction was detected in 37% of SLE patients versus 8.7% in controls (*p* = 0.001) [31]. Multivariate analyses confirmed that SLE independently predicted subclinical LV dysfunction (OR 6.07; 95% CI 1.68–21.99), with male sex, obesity, and arterial hypertension further increasing the risk [31].

### Heart failure in rheumatoid arthritis

RA confers a twofold increased risk of congestive HF, primarily due to systemic inflammation [3, 32]. Systematic reviews have demonstrated that RA increases HF risk and elevates all-cause and cardiac mortality, even after percutaneous coronary interventions [3, 33]. In a cross-sectional study of 37,736 US subjects, the prevalence of HF among RA patients was 7.1%, with RA significantly associated with increased HF risk (OR 1.9, 95% CI 1.5–2.5) [34]. Similarly, a retrospective study on 67,850 RA patients reported elevated risks for both HF with preserved ejection fraction (aHR 1.51, 95% CI 1.46–1.57) and reduced ejection fraction (aHR 1.34, 95% CI 1.3–1.38), with higher mortality among seropositive patients (aHR 2.05, 95% CI 1.76–2.39) [35]. HF incidence increased progressively over time, with no significant improvements despite RA management [35].

Cohort studies further illustrate the relationship between RA and HF subtypes. In a cohort of 20,916 patients with newly diagnosed HF, 1.6% had RA compared with 1% among 103,501 HF-free controls [36]. RA was associated with higher odds of both HF with reduced LVEF (< 40%, OR 1.4, 95% CI 1.1–1.8) and preserved LVEF (≥ 40%, OR 1.6, 95% CI 1.3–2) [36]. Elevated all-cause mortality was observed exclusively in RA patients with reduced LVEF (HR 1.4, 95% CI 1.1–1.8) [36].

Population-based studies reinforce the independent contribution of RA to HF risk. In a cohort of 575 RA patients and 583 controls, HF incidence was 1.99 versus 1.16 per 100 person-years (rate ratio 1.7, 95% CI 1.3–2.1), with a 30-year cumulative incidence of 34% versus 25.2% (*P* < 0.001) [37]. Adjusted analyses demonstrated that RA conferred an 87% higher hazard of HF (HR 1.87, 95% CI 1.47–2.39), with the highest risk among seropositive patients (HR 2.6, 95% CI 1.95–3.43) [37]. The excess risk persisted independently of traditional cardiovascular risk factors and ischemic heart disease [37]. Importantly, HF in RA predominantly manifests as HFpEF. Cohort studies indicate that RA is associated with a twofold increase in the risk of HFpEF (HR = 2; 95% CI, 1.43–2.77) [38]. Elevated baseline inflammatory burden (ESR/CRP) predicts HF risk at both 5 years (HR 1.66, 95% CI 1.12–2.46) and 10 years (HR 1.46, 95% CI 1.13–1.9), primarily driven by HFpEF (5-year HR 1.72; 10-year HR 1.45) [39].

Nationwide cohort studies further corroborate RA as an independent risk factor for HF [40, 41]. In Denmark, among 4.3 million adults, RA patients exhibited a 30% higher risk of HF hospitalization after adjustment for confounders (IRR 1.3, 95% CI 1.17–1.45) [40]. Likewise, the Korean NHIS-HEALS cohort demonstrated that seropositive RA conferred a 2.5-fold increased risk of HF over 12 years (95% CI 1.45–4.3), particularly among older women and individuals without traditional cardiovascular risk factors [41].

The elevated cardiovascular risk in RA arises from the interplay between traditional risk factors, persistent systemic inflammation, and disease-specific mechanisms, including inflammatory dyslipidemia and endothelial dysfunction [42]. Suppression of inflammation by conventional disease-modifying anti-rheumatic drugs (DMARDs) and emerging targeted therapies partially mitigates HF risk. However, optimal management of traditional cardiovascular risk factors remains unanswered [42]. Biological DMARD therapy has been shown to reduce the incidence of HF (OR = 0.84; 95% CI, 0.74–0.95) and all-cause mortality (OR = 0.64; 95% CI, 0.58–0.7), with the greatest benefit observed with TNF-α inhibitors [43].

Non-pharmacologic interventions also provide cardiovascular benefits [44]. Structured aerobic and resistance training improves arterial stiffness, ventricular relaxation, and ventricular–arterial coupling in RA patients, underscoring the potential of lifestyle-based strategies to mitigate cardiovascular risk [44]. Biomarkers such as NT-proBNP offer sensitive measures of cardiac strain and correlate with inflammatory markers, although standard antirheumatic therapies do not consistently reduce HF incidence [45].

Genetic and epidemiological studies further support the link between RA and HF. Mendelian randomization analyses indicate that genetic susceptibility to RA modestly increases HF risk (OR = 1.022, 95% CI 1.005–1.039), independent of NT-proBNP levels [46]. In contrast, genome-wide analyses encompassing 95,524 HF cases and 1,270,968 controls found no direct causal effect of genetically predicted RA on HF, suggesting that RA-targeted therapies alone may not fully mitigate HF risk [47].

### Heart failure in systemic sclerosis

Myocardial perfusion defects and chronic inflammation drive fibrosis, resulting in structural remodeling, arrhythmias, and HF in systemic sclerosis (SSc). Early recognition and intervention are therefore essential, with emerging therapies targeting microvascular injury, myocardial inflammation, and fibrotic remodeling [48].

Cardiac involvement in SSc is often subclinical [49]. In a cross-sectional study on 61 patients (92% female, mean age 63 years), 48% presented with preclinical HF and 38% had symptomatic HF [49]. Advanced HF was associated with older age and multiorgan involvement [49].

Of 4.7 million HF hospitalizations in 2016–2019, 8,150 (0.17%) had SSc [50]. Despite fewer traditional cardiovascular risk factors, SSc patients had higher rates of interstitial lung disease (23.1% vs. 2%; *P* < 0.01) and pulmonary hypertension (36.6% vs. 12.7%; *P* < 0.01) [50]. In-hospital mortality was significantly higher among SSc patients (5.1% vs. 2.6%; aOR 1.81, 95% CI 1.44–2.28; *P* < 0.001) [50].

HF with preserved EF is both frequent (27%) and prognostically relevant in SSc [51]. Such patients are older and have more cardiovascular comorbidities [51]. Over a median nine-year follow-up, HF with preserved EF is associated with a 3.4-fold higher mortality risk (HR 3.4, 95% CI 1.21–9.31) [51].

In a cohort study on 72 SSc patients, 21 had elevated NT-proBNP levels (> 220 pg/ml), reflecting increased left ventricular filling pressures [52]. In 684 SSc patients, myocardial inflammation was detected by cardiac MRI in 74.3% of patients at baseline [53]. After 12 months of immunosuppressive therapy, NT-proBNP, high-sensitivity troponin T, and CRP significantly decreased [53]. Elevated cardiac markers, such as cTn and BNP/NT-proBNP, are frequently associated with echocardiographic abnormalities, arrhythmias, and HF in SSc [54].

An analysis of EUSTAR cohort (*n* = 5,741) demonstrated that SSc with primary heart involvement is associated with markedly reduced survival (*P* < 0.0001) [55]. Incident SSc with primary heart involvement was predicted by telangiectasia, gastrointestinal involvement, age, and anti-topoisomerase I antibodies [55].

Immunosuppressive therapy remains central to controlling myocardial inflammation, while emerging antifibrotic strategies hold promise for mitigating progressive remodelling in SSc [56]. An integrated approach combining early detection, targeted immunomodulation, and antifibrotic therapy may optimize outcomes in patients with SSc-related cardiac disease [56].

HF in SSc may also require intensive care and even referral to extracorporeal membrane oxygenation and heart transplantation [57].

### Heart failure in Sjögren syndrome

Primary Sjögren syndrome (PSS) exerts heterogeneous effects on cardiovascular risk, ranging from subtle changes to severe cardiac affections [58, 59]. In a Danish cohort of 5,092 PSS patients and 20,368 matched controls, the 10-year incidence of HF was 4% versus 2.8%, respectively, with PSS conferring a 42% higher HF risk (HR 1.42, 95% CI 1.2–1.68) [58]. By contrast, a nationwide Taiwanese cohort study on 16,466 matched pairs observed no significant increase in HF risk (10-year cumulative incidence 4.33%; HR 0.98, 95% CI 0.84–1.14) [59].

Sporadically, HF manifests as the initial presentation of PSS [60]. In fact, a 70-year-old patient with PSS without traditional cardiovascular risk factors developed HF, confirmed via autoimmune antibody testing and labial gland biopsy; combined standard HF and immunosuppressive therapy prevented HF readmissions at one-year follow-up [60].

### Heart failure in ankylosing spondylitis

Population-based evidence suggests that AS independently increases the risk of HF and mortality [61]. In a Korean cohort of 12,988 AS patients and 64,940 matched controls, HF incidence was significantly higher in AS patients (0.79% vs. 0.32%, *p* < 0.0001), as was all-cause mortality (1.62% vs. 0.98%, *p* < 0.0001) [61]. Adjusted analyses confirmed a 2.28-fold increase in the risk of HF (95% CI 1.8–2.89) and a 1.66-fold increase in mortality (95% CI 1.42–1.95) [61].

Subclinical cardiac dysfunction is prevalent in AS, and it affects both systolic and diastolic functions [62]. A systematic review of 30 case-control studies (*n* = 2,933) reported lower EF in AS [62]. Valvular abnormalities are also frequent in AS [63]. A cross-sectional study of 4,082 AS patients revealed increased prevalence of aortic stenosis (OR 2.25; 95% CI 1.57–3.23), aortic insufficiency (OR 2.44; 95% CI 1.5–3.94), and mitral insufficiency (OR 1.75; 95% CI 1.17–2.61) [63].

Drug therapies in AS influence the incidence of HF [64]. A Korean cohort study on 19,775 AS patients reported that cumulative NSAID exposure was associated with a dose-dependent increase in HF (aHR 1.12; 95% CI 1.08–1.16) [64].

### Heart failure in idiopathic inflammatory myopathy

Polymyositis and dermatomyositis (PM/DM) are associated with a markedly increased risk of HF and adverse post-HF outcomes [65]. In a Taiwanese cohort study on 2,025 PM/DM patients, the 10-year cumulative incidence of HF was 7.4% [66]. After multivariable adjustment, PM/DM was linked to a significantly higher HF risk (aHR 3.29; 95% CI 2.6–4.18) [65]. Likewise, in a Danish cohort of 936 DM/PM patients compared with 3,744 controls, 10-year HF incidence was higher in patients with DM/PM (6.98%; 95% CI 5.16–9.16), and HF was associated with increased mortality (HR 1.58; 95% CI 1.01–2.47) [67].

Cardiac involvement in PM/DM often begins subclinically [68]. In a two-year prospective study of 28 idiopathic inflammatory myopathy patients without baseline cardiac symptoms, those with a polyphasic disease course exhibited significant declines in left ventricular EF (51.7 ± 0.7% vs. 62.6 ± 0.6%) [68]. Tissue Doppler imaging revealed early right ventricular systolic dysfunction [68]. Diastolic dysfunction frequently progresses [69].

Finally, a systematic review of 13 studies pooling 391 adult patients with idiopathic inflammatory myopathy, 227 juvenile dermatomyositis patients, and 550 controls confirmed impaired ventricular relaxation and elevated filling pressures in idiopathic inflammatory myopathy, highlighting the use of early echocardiographic assessment to reveal subclinical left ventricular diastolic dysfunction and mitigate subsequent HF risk [70].

**Fig. 1:**
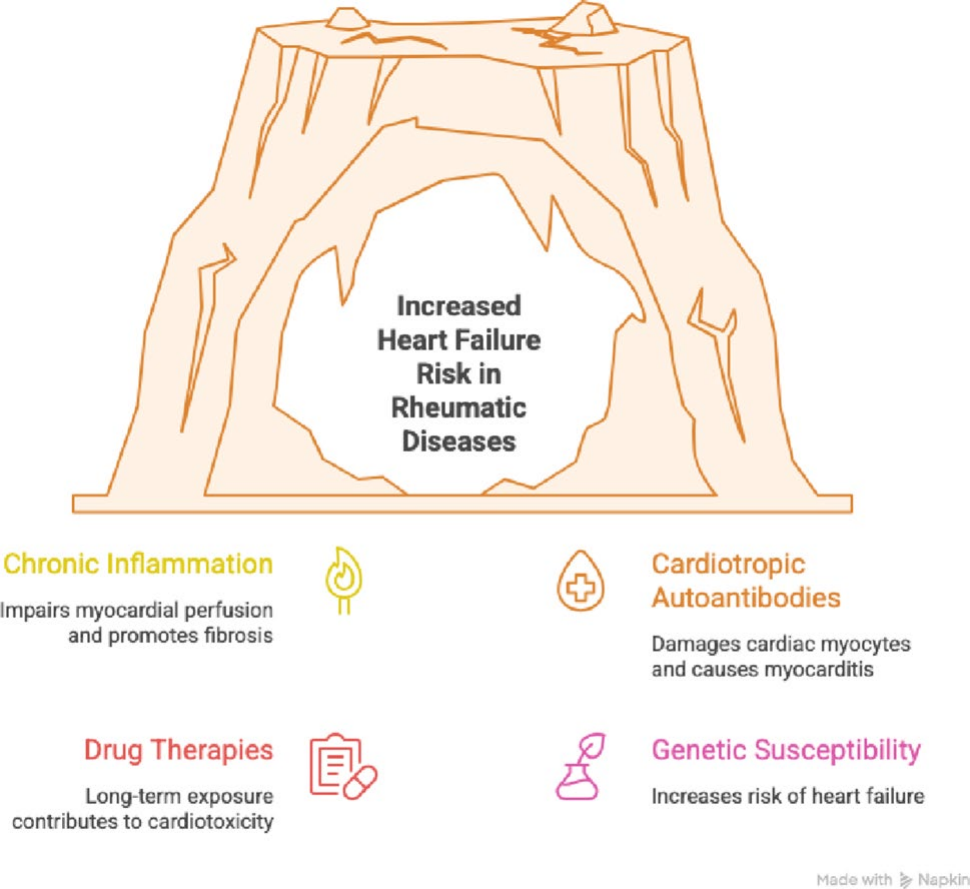
Key risk determinants of heart failure in patients with rheumatic diseases

The main inflammatory, immune-mediated, and treatment-related risk factors contributing to HF in rheumatic diseases are depicted in Fig. 1. The figure provides a concise overview of how systemic inflammation, autoantibody-mediated myocardial injury, and treatment-related factors collectively contribute to the development and progression of HF across autoimmune rheumatic conditions.

### Screening, monitoring, and risk stratification

Early detection of at-risk patients and prompt monitoring are crucial for better outcomes. Clinicians should keep a high level of suspicion, especially in patients with long-standing or severe disease, and perform baseline cardiovascular assessments, including clinical evaluation and standard echocardiography, to identify subclinical myocardial involvement [71, 72]. Ongoing monitoring should include both traditional cardiac biomarkers, including natriuretic peptides, and, when available, advanced imaging techniques, such as speckle-tracking echocardiography or cardiac magnetic resonance [28, 73]. These tools may help to detect early functional or structural cardiac changes before overt HF develops. Risk stratification should combine conventional cardiovascular risk factors with disease-specific parameters, including disease activity, autoantibody profiles, and cumulative exposure to cardiotoxic therapies. Employing structured strategies facilitates customized follow-up and prompt intervention in high-risk patients. Collaboration between rheumatology and cardiology teams is strongly recommended to optimize management, prevent progression to overt HF, and ensure comprehensive care.

## Conclusions

HF is often an underrecognized complication of rheumatic diseases, driven by systemic inflammation, autoimmunity, and disease-specific cardiac affections. Diastolic dysfunction is particularly prevalent, often representing an early and subclinical manifestation of myocardial impairment even in the presence of preserved systolic function. This dysfunction frequently progresses, paralleling disease activity and multisystem involvement. The pattern of cardiac risk varies across rheumatic conditions: SLE and SSc predominantly exhibit diastolic dysfunction with preserved systolic function; AS is characterized by combined systolic and valvular abnormalities; and PM/DM may progress to biventricular impairment. Timely recognition of diastolic abnormalities is paramount, as they frequently precede overt HF and provide a critical window for intervention. Improving outcomes in this high-risk population requires early detection, judicious immunomodulatory therapy, and comprehensive cardiovascular management, underscoring the importance of integrated care by rheumatology and cardiology teams.
